# DNA double strand break repair defect and sensitivity to poly ADP-ribose polymerase (PARP) inhibition in human papillomavirus 16-positive head and neck squamous cell carcinoma

**DOI:** 10.18632/oncotarget.4863

**Published:** 2015-08-22

**Authors:** Alice N. Weaver, Tiffiny S. Cooper, Marcela Rodriguez, Hoa Q. Trummell, James A. Bonner, Eben L. Rosenthal, Eddy S. Yang

**Affiliations:** ^1^ Department of Radiation Oncology, University of Alabama at Birmingham, Birmingham, AL 35249, USA; ^2^ Department of Surgery, University of Alabama at Birmingham, Birmingham, AL 35249, USA; ^3^ Department of Cell, Developmental, and Integrative Biology, University of Alabama at Birmingham, Birmingham, AL 35249, USA; ^4^ Department of Pharmacology and Toxicology, University of Alabama at Birmingham, Birmingham, AL 35249, USA

**Keywords:** HPV, HNSCC, DNA repair, PARP inhibition

## Abstract

Patients with human papillomavirus-positive (HPV+) head and neck squamous cell carcinomas (HNSCCs) have increased response to radio- and chemotherapy and improved overall survival, possibly due to an impaired DNA damage response. Here, we investigated the correlation between HPV status and repair of DNA damage in HNSCC cell lines. We also assessed *in vitro* and *in vivo* sensitivity to the PARP inhibitor veliparib (ABT-888) in HNSCC cell lines and an HPV+ patient xenograft. Repair of DNA double strand breaks (DSBs) was significantly delayed in HPV+ compared to HPV− HNSCCs, resulting in persistence of γH2AX foci. Although DNA repair activators 53BP1 and BRCA1 were functional in all HNSCCs, HPV+ cells showed downstream defects in both non-homologous end joining and homologous recombination repair. Specifically, HPV+ cells were deficient in protein recruitment and protein expression of DNA-Pk and BRCA2, key factors for non-homologous end joining and homologous recombination respectively. Importantly, the apparent DNA repair defect in HPV+ HNSCCs was associated with increased sensitivity to the PARP inhibitor veliparib, resulting in decreased cell survival *in vitro* and a 10–14 day tumor growth delay *in vivo*. These results support the testing of PARP inhibition in combination with DNA damaging agents as a novel therapeutic strategy for HPV+ HNSCC.

## INTRODUCTION

Head and neck squamous cell carcinomas (HNSCC) are typically aggressive cancers with high recurrence rates and poor 5-year survival. However, patients with human papilloma virus-positive (HPV+) HNSCCs, especially those with oropharyngeal disease (OPSCC), have substantially better outcomes compared to their HPV− counterparts [1–4]. HPV+ OPSCCs demonstrate increased sensitivity to DNA damaging agents such as radiation (IR) and platinum-based chemotherapy compared to HPV− disease [5]. HPV is less commonly found in HNSCCs arising from the oral cavity, hypopharynx, and larynx, and its effect on clinical outcomes in these patients in less clear [6–8]. The enhanced IR sensitivity of HPV+ HNSCCs has been replicated *in vitro* and corresponds with delayed resolution of the DNA double strand break (DSB) marker phosphorylated Histone 2AX (γH2AX) following IR [9, 10]. Although persistence of γH2AX foci in HPV+ HNSCCs is thought to be the result of defective DNA repair, the mechanisms underlying this defect have not been well characterized. Nevertheless, these observations have resulted in the design of clinical trials for de-escalated or targeted therapy in HPV+ patients in order to avoid unnecessary treatment-associated morbidity [11, 12].

Inhibitors of poly-ADP ribose polymerase (PARP) are one class of targeted therapy shown to be effective for tumors with DNA repair deficits [13]. These agents demonstrate synthetic lethality with inherent or induced defects in homologous recombination repair (HR), such as loss of Breast Cancer 1 and 2 (BRCA1/2) protein function, and have recently been approved for use in advanced ovarian cancers with a “BRCAness” phenotype. Our lab has previously shown HPV− HNSCCs to be DNA repair proficient and insensitive to PARP inhibition alone, but more recent work suggests *in vitro* sensitivity to this targeted therapy is increased in HPV+ HNSCC cell lines [14, 15].

Based on these intriguing observations, we performed an in-depth analysis of DNA DSB repair in HPV+ HNSCCs and further investigated the sensitivity of these tumors to PARP inhibition. Here, we report HPV+ HNSCC cell lines have decreased activity of two major DSB repair pathways, HR and canonical non-homologous end joining (NHEJ), leading to a significant delay in the resolution of IR-induced DSBs. Interestingly, HPV+ HNSCCs retain their ability to sense DNA damage, as γH2AX, 53 binding protein 1 (53BP1), and BRCA1 are all recruited to sites of damage. Instead, the deficiency in DNA repair is associated with a loss of DNA-dependent protein kinase (DNA-Pk) and BRCA2 activation following IR and a significant reduction in DNA-Pk and BRCA2 protein levels as compared to HPV− HNSCC. Importantly, these findings correlate with increased sensitivity to PARP inhibition both *in vitro* and *in vivo*, including a patient-derived HPV+ xenograft model. Our results indicate HPV+ HNSCCs have a significant defect in DNA repair that can be exploited with PARP inhibition, which could be combined with other DNA damaging agents to improve the treatment of this disease.

## RESULTS

### HPV+ HNSCCs detect IR-induced DNA double strand breaks but have delayed resolution of damage

Previous studies suggest DNA DSBs persist in HPV+ HNSCCs following ionizing radiation (IR) [9], which may explain the increased radiosensitivity observed in patients with HPV+ as compared to HPV− head and neck cancer. However, the mechanism responsible for DSB persistence has not been fully characterized. We previously reported robust DNA repair, both NHEJ and HR, in the HPV− HNSCC cell lines UM-SCC1, UM-SCC6, and FaDu [14]. So, to investigate differences in DNA repair capacity between HPV+ and HPV− HNSCCs, we used the HPV16+ UM-SCC47 and UPCI:SCC154 HNSCC cell lines, as well as UM-SCC1 cells as a representative HPV− control.

**Figure 1:**
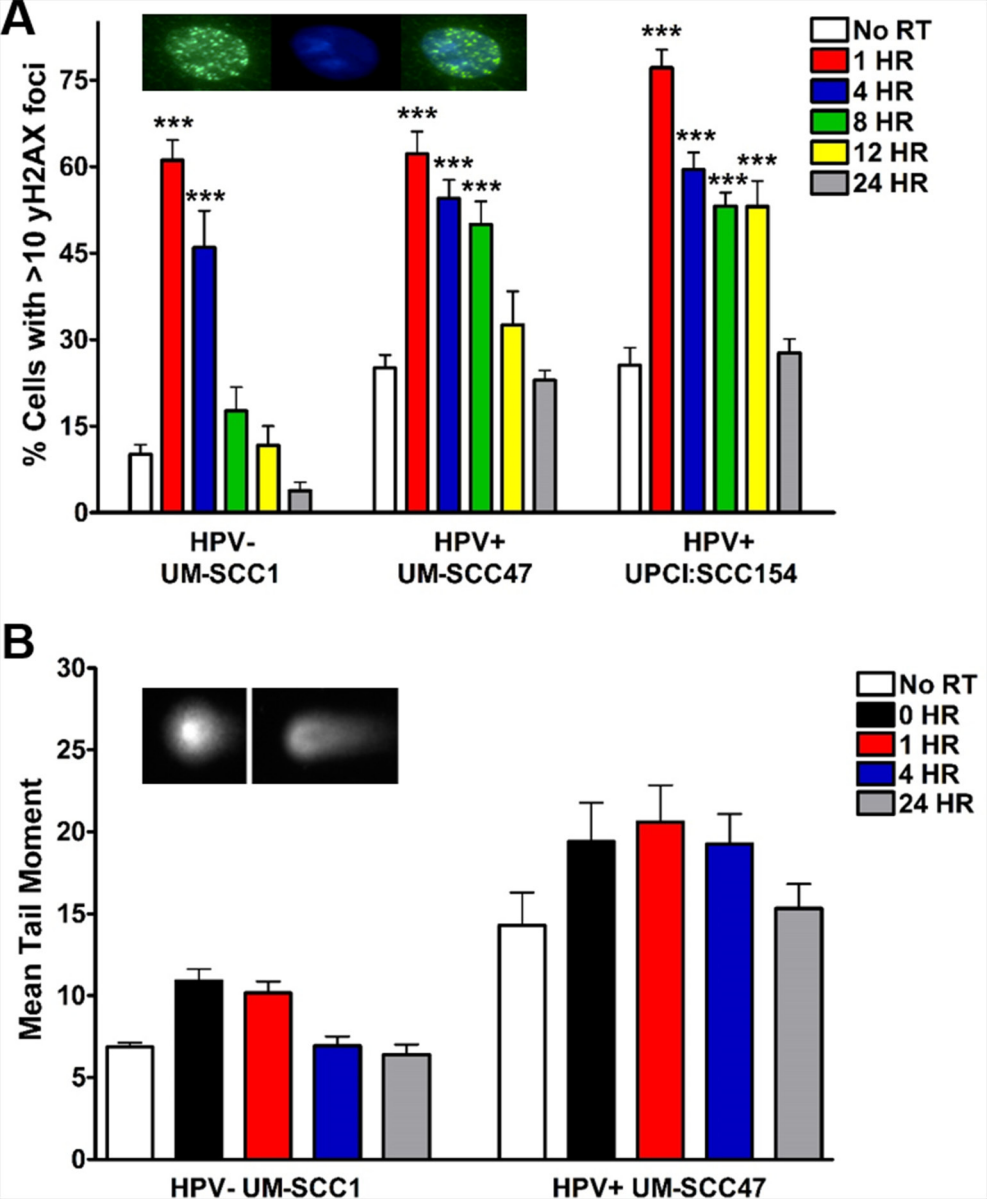
HPV+ HNSCCs exhibit delayed resolution of IR-induced DNA DSBs Cells were subjected to 4 Gy IR and, at the indicated time points, processed for **A.** immunofluorescent staining for γH2AX foci or **B.** neutral comet assay. Inset panels indicate representative images for (A) γH2AX (left), DAPI (middle), and merge (right), and (B) minimal comet tail (left) and positive comet tail (right). Shown is representative data of 2 independent experiments performed in triplicate with mean +/− SEM, with IR groups compared to no IR controls for each cell line. ***p < 0.001, **p < 0.01, *p < 0.05.

First, we measured the kinetics of IR-induced DSB resolution via immunofluorescent staining for the DSB marker γH2AX. In the absence of DNA damage, the percentage of γH2AX foci-positive cells was elevated in HPV+ UM-SCC47 and UPCI:SCC154 cells (25%) as compared to HPV− UM-SCC1 cells (<15%) (Figure 1A, Supplementary Table S1) [14, 16, 17]. All three cell lines displayed a robust increase in γH2AX foci-positive cells following IR, peaking at 1 hour (Figure 1A). Resolution of foci-positive cells was significantly delayed in both HPV+ cell lines, occurring 12–24 hours after IR as compared to 4 hours in HPV− cells (Figure 1A). As confirmation of delayed damage resolution in HPV+ HNSCC cells, we also measured IR-induced DNA DSBs using the neutral comet assay, a single cell gel electrophoresis assay to detect relative amounts of DNA strand breaks [18, 19]. In agreement with γH2AX foci staining, the baseline mean comet tail moment was 2-fold higher in HPV+ as compared to HPV− cells (Figure 1B). IR induced an increase in mean comet tail moment in both HPV− and HPV+ cells, which peaked at the immediate collection and 1 hour time points, respectively (Figure 1B). Damage was resolved by 4 hours in HPV− cells but was delayed until 24 hours in HPV+ cells (Figure 1B). These results indicate that both HPV+ and HPV− HNSCC cells experience an increase in DNA DSBs following IR and generate the γH2AX signal thought to initiate the DNA damage response. However, HPV+ cells experience a significant delay in damage resolution as compared to HPV− counterparts [20].

### NHEJ repair activity and DNA-Pk recruitment are decreased in HPV+ HNSCCs

**Figure 2:**
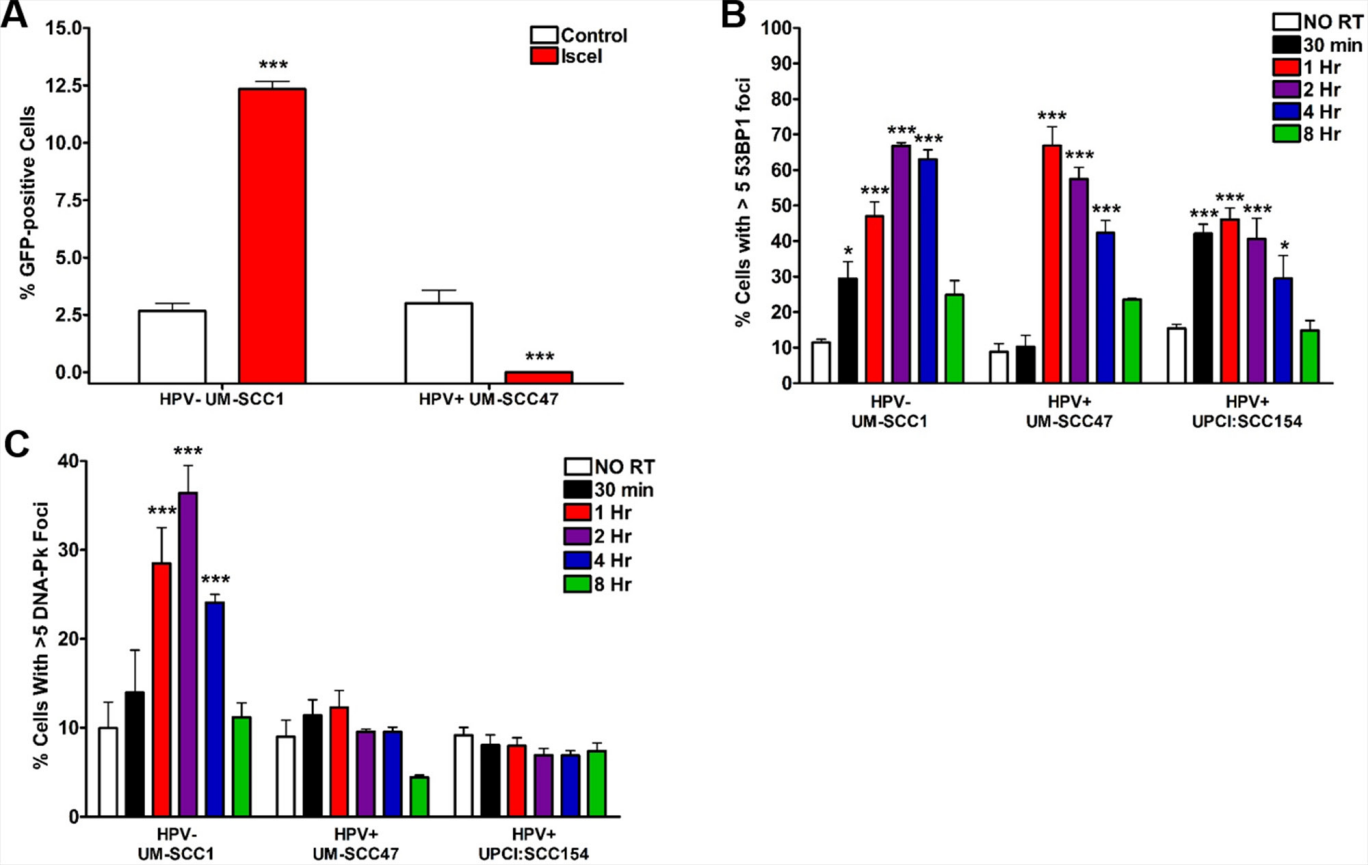
HPV+ HNSCCs harbor defects in NHEJ repair signaling **A.** Chromosomal canonical end joining repair capacity was directly measured in UM-SCC1 and UM-SCC47 cells stably expressing the NHEJ-GFP repair substrate. 48 hours following transfection with ISce-1 or control vector, cells were subjected to flow cytometry for GFP expression. Shown is representative data of 2 independent experiments performed in triplicate with mean +/− SEM, comparing Isce1 groups to empty vector controls. Cells were subjected to 4 Gy IR and, at the indicated time points, processed for immunofluorescent staining for IR-induced **B.** 53BP1 or **C.** pDNA-Pk foci. Shown is representative data of 2 independent experiments performed in triplicate with mean +/− SEM, with IR groups compared to no IR controls for each cell line. ***p < 0.001, **p < 0.01, *p < 0.05.

To determine the mechanism responsible for persistence of DSBs in HPV+ HNSCCs, we first evaluated canonical NHEJ, the primary repair pathway for resolution of IR-induced DSBs. We directly measured NHEJ activity using a GFP-based chromosomal repair assay in UM-SCC1 and UM-SCC47 cells with stable expression of the NHEJ-GFP repair substrate [21], where the percent of GFP-positive cells following endonuclease transfection indicates NHEJ-mediated repair. HPV− UM-SCC1 cells demonstrated a 5-fold increase in GFP-positive cells following endonuclease treatment, indicating active NHEJ-mediated repair (Figure 2A). In stark contrast, the percentage of HPV+ UM-SCC47 cells expressing GFP decreased from baseline after endonuclease exposure (Figure 2A). This decrease may have been a result of cell death, as nonviable cells were excluded from observation.

Next, we examined IR-induced aggregation of 53BP1, an early marker of NHEJ pathway choice [22]. As seen in Figure 2B, both HPV+ and HPV− cell lines demonstrate a significant increase in 53BP1 foci-positive cells following IR, peaking at 1–2 hours and resolved by 8 hours. These results indicate NHEJ pathway choice is intact in HNSCC cells. Then, we evaluated the subsequent recruitment of phosphorylated DNA-dependent protein kinase (DNA-Pk), a serine/threonine kinase whose function is necessary for execution and completion of NHEJ repair. HPV− HNSCC cells exhibit an 4-fold increase in pDNA-Pk foci-positive cells after IR, peaking at 40% at 2 hours (Figure 2C). Interestingly, no significant increase in pDNA-Pk foci-positive cells was observed in either HPV+ HNSCC cell line (Figure 2C). Our results suggest that although initiation of NHEJ repair is intact in HPV+ HNSCCs, decreased recruitment of the important downstream repair factor DNA-Pk severely limits NHEJ-mediated damage resolution.

### HPV+ HNSCCs activate HR but have decreased recruitment of BRCA2

**Figure 3:**
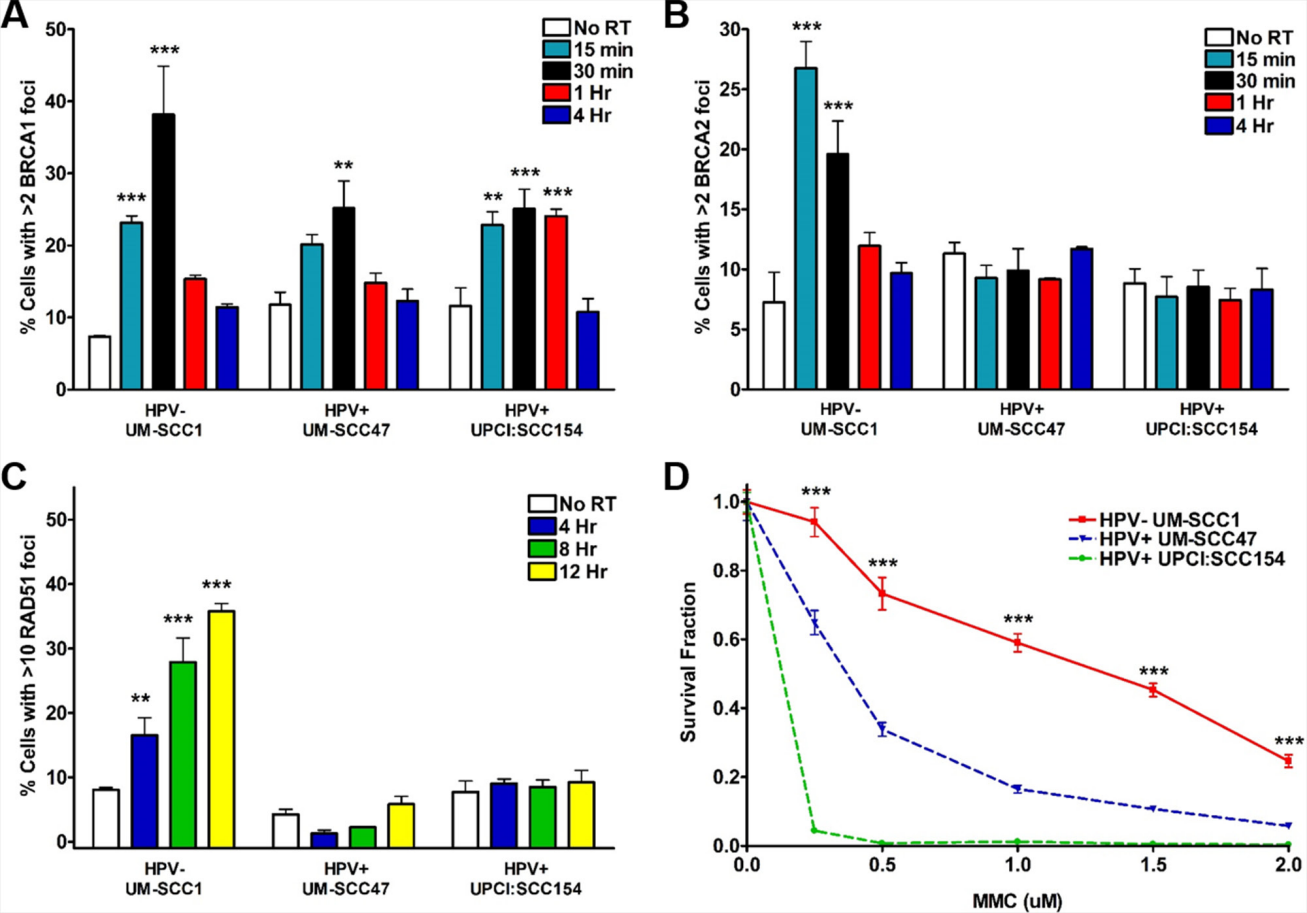
HPV+ HNSCCs display defects in HR repair signaling Cells were subjected to 4 Gy IR and, at the indicated time points, processed for immunofluorescent staining for IR-induced **A.** BRCA1, **B.** BRCA2, or **C.** RAD51 foci. Shown is representative data of 2 independent experiments performed in triplicate with mean +/− SEM, with IR groups compared to no IR controls for each cell line. **D.** Cells were plated at two different densities, treated with increasing doses of mitomycin C, left undisturbed for approximately 2 weeks, then fixed and stained for colony counting. Shown is representative data of 2 independent experiments performed in triplicate with mean +/− SEM comparing HPV− to HPV+ cell lines. ***p < 0.001, **p < 0.01, *p < 0.05.

IR-induced DSBs can also be repaired through HR, although activity of this pathway is limited to S- and G2-phase cells. To interrogate the HR pathway, we initially examined the ability of HPV+ and HPV− HNSCCs to form BRCA1 foci, the first step in HR repair signaling [22]. All three cell lines demonstrated a significant IR-induced increase in BRCA1 foci-positive cells, with a peak at 30 minutes and return to baseline at 1–4 hours (Figure 3A). We then evaluated the next two steps in the HR pathway, recruitment of BRCA2 and, subsequently, RAD51 to sites of DNA damage. Following IR, the percentage of foci-positive HPV− HNSCC cells increased robustly for both BRCA2 (4-fold increase at a peak of 15 minutes) and RAD51 (4.5-fold increase at a peak of 12 hours) (Figure 3B, 3C). In contrast, IR-induced BRCA2 and RAD51 foci were strikingly absent in HPV+ cell lines (Figure 3B, 3C). As attempts to assess HR pathway activity via a GFP-based chromosomal repair assay were unsuccessful, we examined the sensitivity of HNSCCs to mitomycin C (MMC)-induced interstrand crosslinks, a type of DNA damage specifically repaired through the HR pathway, as further indication of an HPV-associated HR defect [23–26]. We observed significantly lower cell survival in HPV+ as compared to HPV− cells at all doses of MMC (Figure 3D), suggesting HPV+ cells are unable to repair crosslinking damage. Taken together, these results support the initial activation of HR in HPV+ HNSCCs but an inability to recruit BRCA2 and RAD51 to DSBs resulting in a HR defect.

### Protein expression of DNA-Pk and BRCA2 is reduced in HPV+ HNSCCs

**Figure 4:**
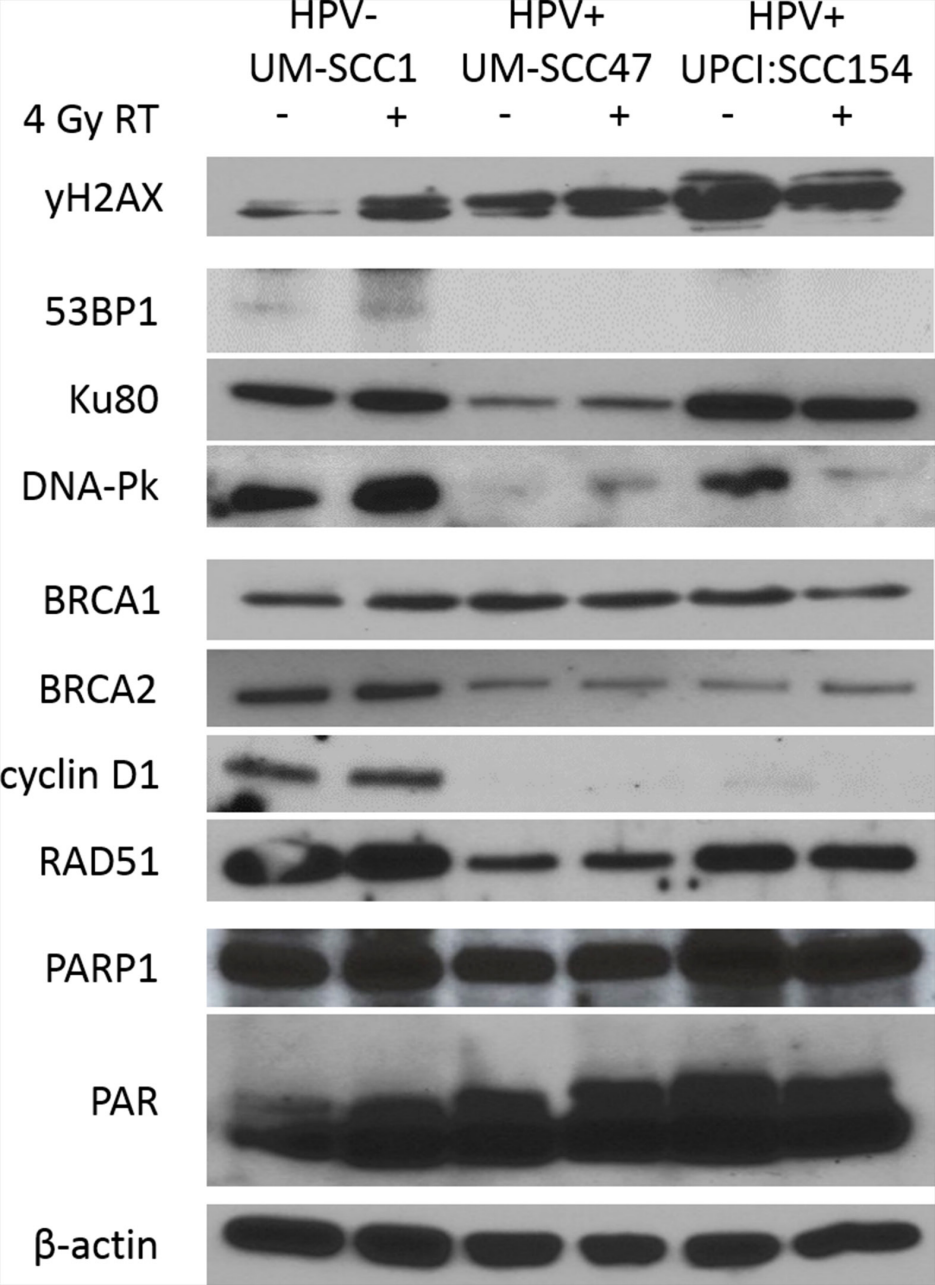
HPV+ HNSCCs have decreased expression of NHEJ and HR proteins including DNA-Pk and BRCA2 Cells were treated with mock or 4 Gy radiation, harvested and lysed at 15 minutes post-treatment, and analyzed by western blot for relative expression of indicated proteins. β-actin was used as a loading control. Shown is a representative blot from 2 independent experiments.

Having observed a defect in DNA-Pk and BRCA2 protein activity in response to DNA damage in HPV+ HNSCC cells, we hypothesized that expression of these proteins may be diminished as well. As assessed by SDS-PAGE, HPV+ HNSCC cells had significantly decreased expression of the NHEJ proteins 53BP1 and DNA-Pk as compared to HPV− cells (Figure 4). Expression of HR proteins BRCA2 and cyclin D1 was also markedly reduced in HPV+ compared to HPV− cells, in addition to a slight decrease in RAD51 expression (Figure 4). To determine the level of regulation at which differential expression occurs, we examined mRNA expression of DSB repair genes using the NanoString nCounter platform, a multiplexed digital gene expression system which uses molecular barcodes to detect and count unique transcripts in a single hybridization reaction and which has been validated by other techniques such as mRNA-seq, microarray, and qRT-PCR [27–32]. Interestingly, differential protein expression of DSB repair factors cannot be explained by mRNA expression levels (Supplementary Figure S1). Only cyclin D1 and RAD51 were found to have corresponding differential expression at the mRNA level, with decreased transcript levels in HPV+ as compared to HPV− HNSCC cells (Supplementary Figure S1). We also evaluated expression of the DNA repair enzyme PARP1 and its product poly (ADP-ribose) (PAR), a surrogate marker for PARP activity and a potential biomarker for sensitivity to DNA repair-targeted therapy with PARP inhibition [33–35]. While expression of PARP1 was similar across all three cell lines and unchanged by IR, PAR expression was marginally elevated in the two HPV+ cell lines and increased with IR (Figure 4). These results indicate the inability of HPV+ HNSCC cells to form DNA-Pk and BRCA2 foci corresponds with decreased expression of these two proteins, potentially accounting for the observed defects in both NHEJ and HR repair and the persistence of DSBs in HPV+ HNSCCs. In addition, HPV+ cells may have increased PARP1 activity, suggesting a means by which this repair defect can be targeted therapeutically.

### Decreased *in vitro* survival of HPV+ HNSCCs treated with the PARP inhibitor veliparib

**Figure 5:**
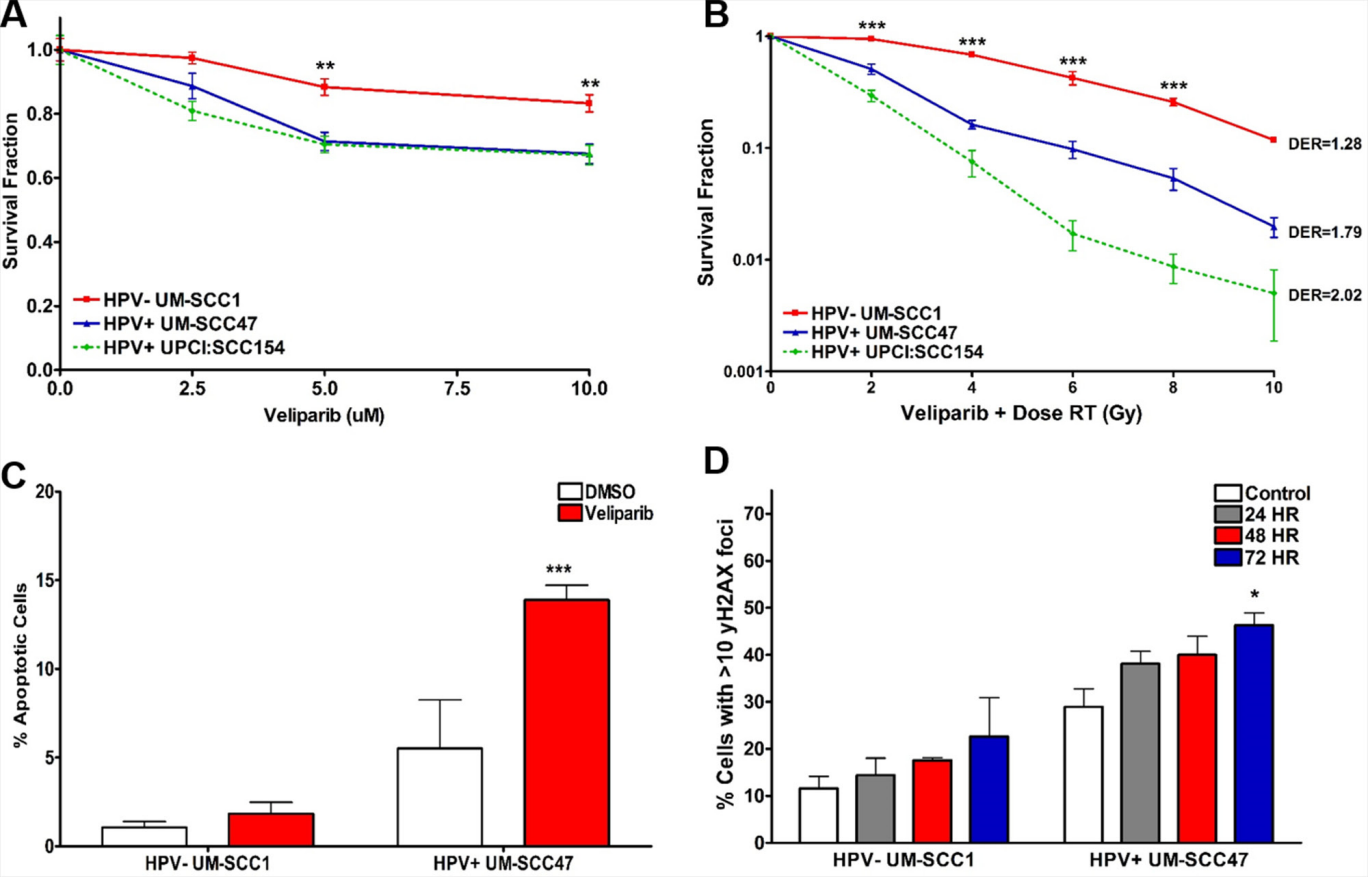
HPV+ HNSCCs are sensitive to PARP inhibition *in vitro* Cells were plated at two different densities and treated with **A.** increasing doses of the PARP inhibitor veliparib or **B.** 10 μM veliparib with increasing doses of IR. After treatment, cells were left undisturbed for approximately 2 weeks, then fixed and stained for colony counting. Dose enhancement ratio (DER) was calculated at 50% survival. **C.** Cells treated with 10 μM veliparib for 72 hours were labeled with Annexin V-FITC and propidium iodide and assessed for apoptosis by flow cytometry. Early and late apoptosis were combined to reflect total population of apoptotic cells. **D.** Cells treated with 10 μM veliparib were processed at the indicated time points for immunofluorescent staining for γH2AX foci. Shown is the mean +/− SEM from at least 2 independent experiments performed in triplicate with (A, B) HPV− compared to HPV+ cell lines or (C, D) treated compared to control groups for each cell line. ***p < 0.001, **p < 0.01, *p < 0.05.

As HPV+ HNSCC cell lines demonstrate delayed DSB repair, decreased NHEJ and HR activity, and slightly increased PARP1 activity, we evaluated the sensitivity of these models to PARP inhibition, a class of targeted therapies shown to be efficacious in HR-deficient tumors [15, 36, 37]. First, we assessed *in vitro* cell survival by colony formation assay in response to treatment with the PARP inhibitor veliparib, which has been proven safe and effective in combination with chemotherapy and radiation in solid tumor clinical trials [38, 39]. Cells were treated with 0–10 μM veliparib, which are known physiologic concentrations achievable in patients. HPV− UM-SCC1 cells exhibited a small but statistically significant decrease in survival fraction at 5 and 10 μM veliparib (Figure 5A). However, HPV+ cell lines were 1.5-fold more sensitive to veliparib than HPV− cells at the same doses (Figure 5A). The increased sensitivity in HPV+ HNSCC cells was magnified when 10 μM veliparib was given in combination with low dose IR (Figure 5B). We also compared the radiosensitizing effects of veliparib to that of the anti-epidermal growth factor receptor (EGFR) monoclonal antibody cetuximab, another targeted agent which modifies DNA DSB repair and is FDA-approved for use in head and neck cancers [14, 40]. Treatment with cisplatin alone was more effective than either veliparib or cetuximab in HPV+ HNSCC cells (data not shown). However, radiosensitivity was similar in HPV+ cells pre-treated with either veliparib, cetuximab, or cisplatin at low doses of radiation (Supplementary Figure S2). We next evaluated the activation of cellular apoptosis in response to veliparib by determining the percentage of cells positive for Annexin V, a cell surface marker which acts as an early indicator of apoptosis. Activation of apoptosis was significantly greater in HPV+ UM-SCC47 cells treated with 10 μM veliparib compared to vehicle (2-fold increase at 72 hours). This effect was not observed in HPV− UM-SCC1 (Figure 5C). To examine the mechanism of toxicity induced by PARP inhibition, we measured the accumulation of γH2AX foci in cells treated with vehicle or a 10 μM dose of veliparib. Consistent with a repair deficiency in HPV+ cells, a statistically significant increase in γH2AX foci-positive cells was observed in HPV+ UM-SCC47 but not HPV− UM-SCC1 cells after 72 hours of veliparib (Figure 5D). Taken together, our results suggest increased *in vitro* sensitivity to PARP inhibition in HPV+ HNSCC cells, which is maximized by the addition of IR. PARP inhibitor sensitivity is associated with the accumulation of DNA damage and increased apoptosis in these cells.

### Veliparib causes *in vivo* tumor growth delay in HPV+ HNSCC cells and a patient-derived xenograft

**Figure 6:**
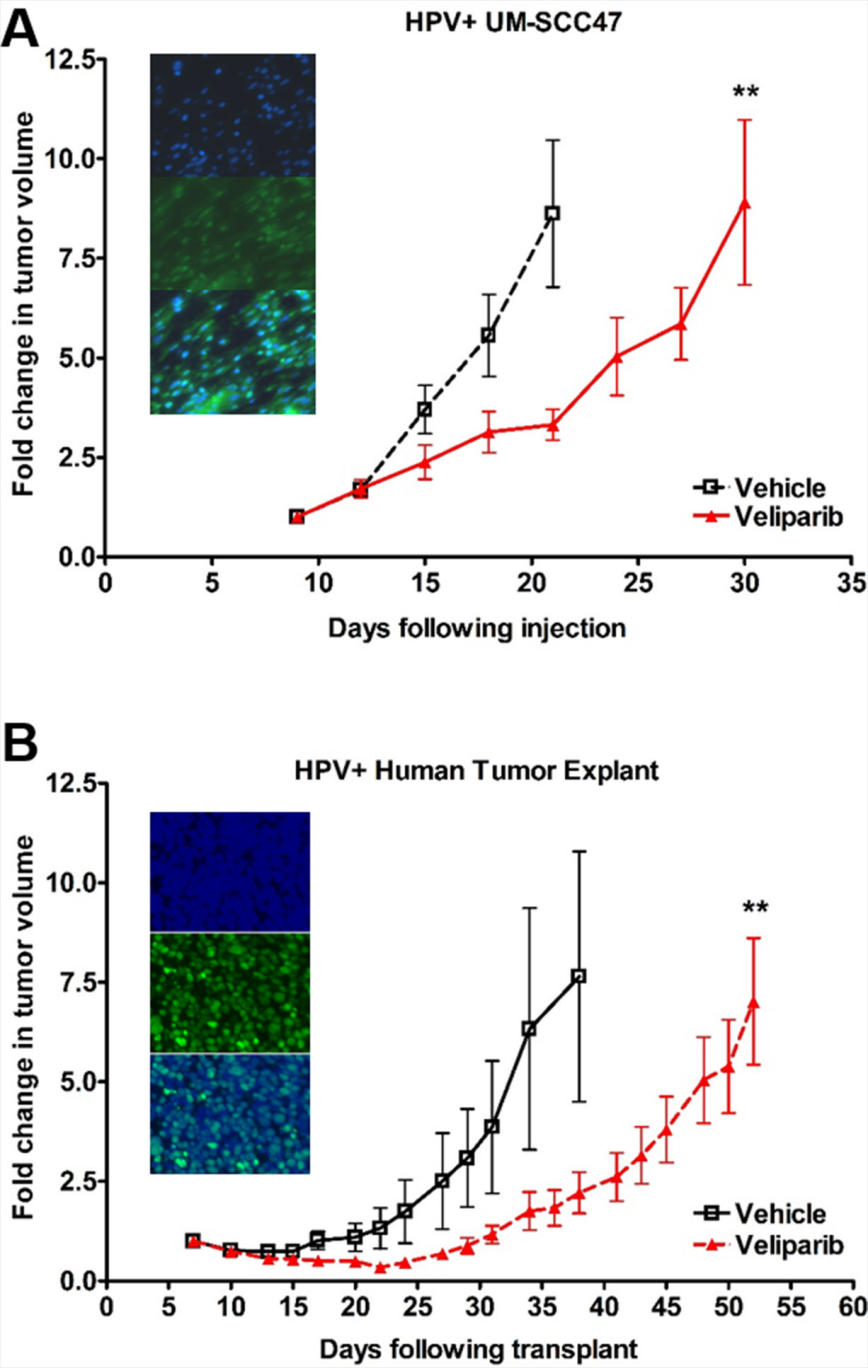
HPV+ HNSCCs are sensitive to PARP inhibition *in vivo* **A.** 5 million UM-SCC47 cells were injected subcutaneously or **B.** patient tumor explants were minced into 1–2 mm slivers and implanted subcutaneously into the right flank of NOD-SCID mice. Once tumors reached 20 mm^3^ by caliper measurement, mice were randomly divided into vehicle (normal saline) or veliparib (200 mg/kg twice daily) groups with 10 mice per group. Mice were treated by oral gavage twice per day, with tumors measured by caliper every 2–3 days. Shown is the mean fold change in tumor volume +/− SEM. **p < 0.01. Inset panels show representative staining images of formalin fixed, paraffin-embedded tumors from mice in the vehicle-treated groups. Slides were imaged for nuclear DAPI (top), p16 (middle), and merge (bottom).

With our *in vitro* studies showing increased sensitivity of HPV+ HNSCC cells to PARP inhibition, we next validated these findings *in vivo* by assessing tumor growth rate in mice bearing HPV+ HNSCC xenografts. We used an HPV+ UM-SCC47 flank xenograft as well as a previously described patient-derived xenograft from an HPV+ HNSCC lymph node metastasis [41] as *in vivo* models. Consistent with our *in vitro* findings, veliparib treatment corresponded to a 10-day growth delay in the UM-SCC47 xenografts (Figure 6A) and, excitingly, a 14-day growth delay in the HPV+ patient-derived tumor xenografts (Figure 6B). Following completion of treatment, tumors were harvested and HPV status was confirmed by positive p16 staining for both models (Figures 6A, 6B inset panels). In contrast, HPV− UM-SCC1 cells were not responsive to PARP inhibition alone *in vivo* (Supplementary Figure S3) [42]. These results indicate *in vivo* susceptibility of HPV+ HNSCC to the PARP inhibitor veliparib.

## DISCUSSION

In this study, we report the nature of a DNA repair defect in HPV+ HNSCCs encompassing both the NHEJ and HR repair pathways. This defect corresponds with decreased protein expression and activity of DSB repair proteins DNA-Pk and BRCA2 in HPV+ HNSCC cell lines. Importantly, we also demonstrate sensitivity of HPV+ HNSCC to the PARP inhibitor veliparib as a single agent both *in vitro* and *in vivo* in cell line and patient-derived tumor xenografts, an effect magnified by low dose IR. These findings provide further insight into the increased sensitivity of HPV+ HNSCC to DNA damaging agents such as radio- and chemotherapy. Furthermore, targeted therapy with PARP inhibition may be used in combination with other DNA damaging agents to better treat this disease.

Recently, a focus has been placed on the role of HPV in DNA damage repair. High risk HPVs express two oncoproteins, E6 and E7, which have a multitude of activities at the molecular level including proteasomal degradation of tumor suppressors p53 and retinoblastoma protein (Rb). p16^INK4A^, which is significantly upregulated by HPV E7, was reported to inhibit HR repair in HPV+ HNSCCs through downregulation of cyclin D1 and decreased RAD51 recruitment [43]. In addition, HPV E7 may directly alter HR repair in HNSCCs through dysregulation of RAD51 expression [44]. HPV E6 has also been implicated as a mediator of DNA repair activity, with E6 expression corresponding to defects in error-free DNA end joining and XRCC1-mediated nucleotide excision repair activity in other models [45, 46]. All of these observations support our findings that HPV+ HNSCCs harbor defects in both NHEJ and HR, although we are the first to implicate BRCA2 and DNA-Pk as potential mediators of defective repair.

Previous studies have shown elevated γH2AX foci in HPV+ HNSCC cell lines following IR [9, 10]. However, these groups also demonstrated a radiation-induced cell cycle arrest, for which γH2AX may be a marker, in the same cell lines [9, 47]. Hence, it has previously been unclear as to the source of elevated γH2AX foci. In this study, we use a combination of γH2AX foci and the neutral comet assay at multiple time points following IR to identify the delayed kinetics of DNA DSB resolution, indicating that DNA repair is, in fact, deficient in HPV+ HNSCCs.

We further evaluated the nature of the DNA repair defect in HPV+ HNSCCs by investigating the components of both primary DSB repair pathways, NHEJ and HR. Surprisingly, 53BP1 and BRCA1, which compete to determine DSB repair pathway choice [22], were both activated by IR in HPV+ HNSCC cells. Although 53BP1 protein expression appears to be diminished in HPV+ cell lines, intact foci formation suggests the quantity is sufficient to respond to DNA damage. In contrast, both protein expression and foci formation of DNA-Pk and BRCA2, key effectors of NHEJ and HR respectively, were strikingly diminished in HPV+ compared to HPV− HNSCCs. Expression of DNA repair proteins has been previously analyzed with respect to HPV status in clinical HNSCC samples, although no consistent patterns have emerged [48, 49]. These inconsistencies may be related to the separation of tumors based on HPV-status alone despite reports indicating HPV+ tumors from patients with a history of smoking behave clinically as HPV− disease [50]. Another potential confounding factor could be the methods and thresholds used for determining HPV status, as these vary between pathologists. Additional studies of protein expression in tumors categorized by both HPV and smoking status are needed to determine the relative influences of smoking and HPV on molecular profile, ultimately leading to more accurate predictions of clinical behavior. Nevertheless, our results suggest the reduced expression of DNA repair proteins may explain the inherent DNA repair defect and increased therapeutic response in this disease.

While we found significantly decreased expression of multiple NHEJ and HR repair proteins in HPV+ HNSCC cell lines, BRCA1 and RAD51, two key HR proteins often identified as the culprits in other HR-defective cancers, appear relatively unaffected. The effects of HPV on cell cycle, and p16^INK4A^ specifically, provide a logical explanation for altered cyclin D1 expression, but it is difficult to conceive of a mechanism that would account for decreased expression of 53BP1, DNA-PK, and BRCA2 without affecting BRCA1 and RAD51. One potential hypothesis may involve the HPV oncoprotein E7, which has been shown to rescue expression of BRCA1 and RAD51 specifically by decreasing the activity of the repressive E2F4 transcription factor, as shown by Hegan et al. [51], without affecting non-E2F regulated repair proteins. Additional studies to further elucidate the source of the protein expression phenotype in HPV+ HNSCCs are needed.

Given the dramatic defects in recruitment of both NHEJ and HR repair proteins following DNA damage, the 2-fold delay in DSB resolution time in HPV+ HNSCCs raises the question of which alternative DSB repair pathways are functional in these cells. Previous reports demonstrate an increase in the alternative NHEJ pathway in the absence of key canonical NHEJ factors, such as Ku86, XRCC4, and DNA ligase IV [52, 53]. While alternative NHEJ has not been fully characterized, it is known to be dependent on activity of PARP1 and DNA ligase III [54, 55]. In this study, HPV+ HNSCCs did not have elevated levels of PARP1, but we did find evidence of increased PARP1 activity. High expression of PAR, in addition to sensitivity to PARP inhibition, could be an indicator of intact PARP1-mediated alternative NHEJ repair. However, additional studies are needed to further evaluate this hypothesis.

DNA repair phenotypes can serve as a potential marker of response to therapy as well as a therapeutic target. PARP inhibitors are a well-tolerated class of DNA repair-targeted agents, which demonstrate significant clinical efficacy in tumors deficient in HR repair [13]. The need for effective treatments with low toxicity profiles is especially apparent in HPV+ HNSCC, as patients are often diagnosed at a relatively young age and may have a prolonged cancer survivorship period. Current therapies can result in debilitating side effects including speech and swallowing dysfunction, xerostomia, PEG tube dependence, cognitive decline, visual impairments, and secondary tumor development, in addition to potential disease recurrence. Here, we demonstrate a significant response to the PARP inhibitor veliparib in both *in vitro* and *in vivo* HPV+ HNSCC models. This response was increased *in vitro* by the addition of IR. Our *in vitro* results are in agreement with a recent publication by Güster et al which showed decreased cell proliferation and increased radiosensitivity in response to the PARP inhibitor olaparib in a panel of HPV+ HNSCC cell lines [15]. Excitingly, we found that veliparib also caused a significant growth delay in a patient-derived explant from a lymph node metastasis of HPV+ HNSCC. These results support further clinical testing of the PARP inhibitor veliparib in HNSCC patients based on HPV and DNA repair status. In addition, other anticancer agents targeting the DNA damage response should be evaluated in HPV+ HNSCC to personalize treatment planning for this disease.

## MATERIALS AND METHODS

### Cell culture and reagents

HPV− HNSCC cell line UM-SCC1 was obtained courtesy of Thomas E Carey, University of Michigan. HPV+ UM-SCC47 and UPCI:SCC154 were a gift from Susan Golin, University of Pittsburg and John H. Lee, Sanford Cancer Research Center. UM-SCC1-luciferase was obtained from Eben Rosenthal, University of Alabama at Birmingham. All cell lines have been previously described and were not further authenticated in our laboratory [16, 17, 56]. HPV-status was confirmed by western blot for p16 expression upon receipt and upon thaw of each plug. UM-SCC1, UM-SCC1-luc, and UM-SCC47 were maintained in DMEM growth medium (Sigma), and UPCI:SCC154 in RPMI growth medium (Gibco). All media were supplemented with 10% fetal bovine serum (SAFC Biosciences) and 1% penicillin/streptomycin (Gibco). UM-SCC1-luc media was also supplemented with 2.0 μg/mL puromycin (Sigma).

### Drugs, plasmids, and transfection

The PARP inhibitor ABT-888 (Enzo Life Sciences *in vitro*, AbbVie *in vivo*) was utilized in our study. Cetuximab (C225, Bristol Myers Squibb), mitomycin C (M4287, Sigma), and cisplatin (CAS 15663-27-1, Tocris Biosciences) were also used. pimEJ5-GFP to measure total chromosomal end joining repair capacity, ISce-1, and empty vectors were gifts from Jeremy Stark, City of Hope, and have been described previously [21]. All transfections were performed using Lipfectamine 3000 (Invitrogen) according to the manufacturer's recommendations.

### Immunofluorescence

Analysis of IR-induced foci was performed as previously described [57]. The following primary antibodies were utilized at manufacturer-recommended dilutions for immunofluorescence: γ-H2AX Ser139 (Cell Signaling, catalog #9718S), BRCA1 (Santa Cruz Biotechnology, catalog #sc-642), BRCA2 H-300 (Santa Cruz Biotechnology, catalog #sc-8326), RAD51 (Santa Cruz Biotechnology, catalog #sc-8349), 53BP1 (Novus Biologicals, catalog #NB100–304), phospho-DNA-Pk Ser2056 (Cell Signaling, catalog #4215). Alexa-fluor 488 conjugated secondary antibodies (Invitrogen) and DAPI (Invitrogen, catalog #D21490) were also used.

### Neutral comet assay

Neutral comet assay was performed using the Trevigen CometAssay Reagent kit as per manufacturer instructions. Cells were visualized using fluorescent microscopy (Carl Zeiss). Images were analyzed using Comet Assay IV System (Perceptive Instruments Ltd, UK).

### Chromosomal repair analysis

UM-SCC1 and UM-SCC47 cells were transfected with EJGFP substrate and stable integrants were selected with 2 μg/mL of puromycin (Sigma) for 3 weeks. Puromycin-resistant colonies were isolated and expanded. Cells were transfected with either empty vector, ISce-1 expression vector to measure repair capacity, or GFP expression vector to measure transfection efficiency. Cells were collecting at the indicated time-points and subjected to two-color fluorescence analysis, which revealed the percentage of GFP+ cells relative to total cell number. 100, 000 cells were processed for each sample. Repair relative to total transfected cells was determined by division of the % GFP+ cells from each ISce-1 transfection by the % GFP+ cells from a parallel transfection.

### Protein expression

Protein was analyzed via SDS-PAGE as previously described [57]. The following primary antibodies were utilized at manufacturer-recommended dilutions for immunoblotting: PARP1 (Santa Cruz Biotechnology, catalog #sc-8007), PAR (Santa Cruz Biotechnology, catalog #sc-56198), ATM (Santa Cruz Biotechnology, catalog #sc-73615), BRCA1 (Santa Cruz Biotechnology, catalog #sc-642), BRCA2 H-300 (Santa Cruz Biotechnology, catalog #sc-8326), RAD51 (Santa Cruz Biotechnology, catalog #sc-8349), 53BP1 (Novus Biologicals, catalog #NB100-304), Ku-86 (Santa Cruz Biotechnology, catalog #sc-9034), DNA-Pk_CS_ C-19 (Santa Cruz, catalog #sc-1552). β-actin (Santa Cruz Biotechnology, catalog #sc-47778) levels were analyzed as a loading control. Species-specific horseradish peroxidase-conjugated secondary antibodies (Santa Cruz Biotechnology) were used at 1:5000 dilution.

### mRNA expression

mRNA was analyzed using the NanoString nCounter platform through the UAB NanoString Laboratory (http://www.uab.edu/medicine/radonc/en/nanostring) [27]. RNA was isolated from cell lines using the Ambion PureLink RNA mini kit (catalog #12183018A). All RNA samples had a concentration > 12.5 ng/μl and an A_260_/A_280_ ratio between 1.7 and 2.3 as determined by DeNovix DS-11 spectrophotometer reading. Samples were then processed for analysis on the NanoString nCounter Flex system using the PanCancer Pathways Plus panel as per manufacturer's instructions. RCC data files were imported into NanoString nSolver 2.5 and normalized as per manufacturer's instructions.

### Colony forming assays

Clonogenic survival was determined by the colony formation assay as previously described [57]. Briefly, cells were seeded, treated with indicated doses of drug, and left undisturbed for two weeks. Cells were then fixed and stained (25% glutaraldehyde, 12 mM crystal violet) and number of colonies (>50 cells) were counted. Survival fraction is equal to (# colonies counted in experimental plate/# cells seeded in experimental plate)/(# colonies counted in control plate/# cells seeded in control plate). Experiments were performed at least in triplicate.

### Apoptosis

Apoptosis was analyzed using the Annexin V-FITC Apoptosis Detection kit (BioVision Research Products, catalog #K101-400) according to manufacturer's instructions and as previously described [57].

### Animal studies

All animal procedures were approved and in accordance with the UAB Institutional Animal Care and Use Committee guidelines. 6–8 week old, 20 g, female NOD-SCID mice (Charles River Laboratories) bearing HNSCC xenografts were treated with 200 mg/kg veliparib twice daily via oral gavage. Mice were inoculated with UM-SCC47 cells and established tumors were measured by caliper 9 days after injection and every 2–3 days thereafter. Mice bearing patient-derived HPV+ tumor xenografts had tumors measured by caliper 7 days after implantation and every 2–3 days thereafter. Mice inoculated with UM-SCC1-luc cells had tumors measured biweekly using a luciferase bioluminescence assay starting at day 7 after implantation.

### Statistical analysis

Unless otherwise specified, data were analyzed via analysis of variance (ANOVA) followed by a Bonferroni post-test using GraphPad Prism version 4.02 (GraphPad Software, San Diego, CA). Data are presented as average +/− standard error of the mean.

## SUPPLEMENTARY FIGURES AND TABLE



## References

[1] Ang KK, Harris J, Wheeler R, Weber R, Rosenthal DI, Nguyen-Tan PF (2010). Human papillomavirus and survival of patients with oropharyngeal cancer. The New England journal of medicine.

[2] Gillison ML, Koch WM, Capone RB, Spafford M, Westra WH, Wu L (2000). Evidence for a causal association between human papillomavirus and a subset of head and neck cancers. Journal of the National Cancer Institute.

[3] Weinberger PM, Yu Z, Haffty BG, Kowalski D, Harigopal M, Brandsma J (2006). Molecular classification identifies a subset of human papillomavirus—associated oropharyngeal cancers with favorable prognosis. Journal of clinical oncology : official journal of the American Society of Clinical Oncology.

[4] Fakhry C, Westra WH, Li S, Cmelak A, Ridge JA, Pinto H (2008). Improved survival of patients with human papillomavirus-positive head and neck squamous cell carcinoma in a prospective clinical trial. Journal of the National Cancer Institute.

[5] Gillison ML, D'Souza G, Westra W, Sugar E, Xiao W, Begum S (2008). Distinct risk factor profiles for human papillomavirus type 16-positive and human papillomavirus type 16-negative head and neck cancers. Journal of the National Cancer Institute.

[6] Chung CH, Zhang Q, Kong CS, Harris J, Fertig EJ, Harari PM (2014). p16 protein expression and human papillomavirus status as prognostic biomarkers of nonoropharyngeal head and neck squamous cell carcinoma. Journal of clinical oncology : official journal of the American Society of Clinical Oncology.

[7] Upile NS, Shaw RJ, Jones TM, Goodyear P, Liloglou T, Risk JM (2014). Squamous cell carcinoma of the head and neck outside the oropharynx is rarely human papillomavirus related. The Laryngoscope.

[8] Annertz K, Rosenquist K, Andersson G, Jacobsson H, Hansson BG, Wennerberg J (2014). High-risk HPV and survival in patients with oral and oropharyngeal squamous cell carcinoma - 5-year follow up of a population-based study. Acta oto-laryngologica.

[9] Rieckmann T, Tribius S, Grob TJ, Meyer F, Busch CJ, Petersen C (2013). HNSCC cell lines positive for HPV and p16 possess higher cellular radiosensitivity due to an impaired DSB repair capacity. Radiother Oncol.

[10] Gupta AK, Lee JH, Wilke WW, Quon H, Smith G, Maity A (2009). Radiation response in two HPV-infected head-and-neck cancer cell lines in comparison to a non-HPV-infected cell line and relationship to signaling through AKT. International journal of radiation oncology, biology, physics.

[11] Ang KK, Zhang QE, Rosenthal DI, Nguyen-Tan PF, Sherman EJ, Weber RS (2011). A randomized phase III trial (RTOG 0522) of concurrent accelerated radiation plus cisplatin with or without cetuximab for stage III-IV head and neck squamous cell carcinomas (HNC). Journal of clinical oncology : official journal of the American Society of Clinical Oncology.

[12] Marur S, Lee J, Cmelak A, Zhao W, Westra WH, Chung CH (2012). ECOG 1308: A phase II trial of induction chemotherapy followed by cetuximab with low dose versus standard dose IMRT in patients with HPV-associated resectable squamous cell carcinoma of the oropharynx (OP). Journal of clinical oncology : official journal of the American Society of Clinical Oncology.

[13] Wielgos M, Yang ES (2013). Discussion of PARP inhibitors in cancer therapy. Pharmaceutical patent analyst.

[14] Nowsheen S, Bonner JA, Lobuglio AF, Trummell H, Whitley AC, Dobelbower MC (2011). Cetuximab augments cytotoxicity with poly (adp-ribose) polymerase inhibition in head and neck cancer. PloS one.

[15] Guster JD, Weissleder SV, Busch CJ, Kriegs M, Petersen C, Knecht R (2014). The inhibition of PARP but not EGFR results in the radiosensitization of HPV/p16-positive HNSCC cell lines. Radiother Oncol.

[16] Ferris RL, Martinez I, Sirianni N, Wang J, Lopez-Albaitero A, Gollin SM (2005). Human papillomavirus-16 associated squamous cell carcinoma of the head and neck (SCCHN): a natural disease model provides insights into viral carcinogenesis. European journal of cancer (Oxford, England : 1990).

[17] Brenner JC, Graham MP, Kumar B, Saunders LM, Kupfer R, Lyons RH (2010). Genotyping of 73 UM-SCC head and neck squamous cell carcinoma cell lines. Head & neck.

[18] Olive PL, Banath JP (2006). The comet assay: a method to measure DNA damage in individual cells. Nat Protoc.

[19] Nowsheen S, Xia F, Yang ES (2012). Assaying DNA damage in hippocampal neurons using the comet assay. J Vis Exp.

[20] Rothkamm K, Lobrich M (2003). Evidence for a lack of DNA double-strand break repair in human cells exposed to very low x-ray doses. Proceedings of the National Academy of Sciences of the United States of America.

[21] Bennardo N, Cheng A, Huang N, Stark JM (2008). Alternative-NHEJ is a mechanistically distinct pathway of mammalian chromosome break repair. PLoS genetics.

[22] Bunting SF, Callén E, Wong N, Chen H-T, Polato F, Gunn A (2010). 53BP1 Inhibits Homologous Recombination in Brca1-Deficient Cells by Blocking Resection of DNA Breaks. Cell.

[23] Hinz JM (2010). Role of homologous recombination in DNA interstrand crosslink repair. Environ Mol Mutagen.

[24] Moynahan ME, Cui TY, Jasin M (2001). Homology-directed dna repair, mitomycin-c resistance, and chromosome stability is restored with correction of a Brca1 mutation. Cancer research.

[25] Birkelbach M, Ferraiolo N, Gheorghiu L, Pfaffle HN, Daly B, Ebright MI (2013). Detection of impaired homologous recombination repair in NSCLC cells and tissues. Journal of thoracic oncology : official publication of the International Association for the Study of Lung Cancer.

[26] Wachters FM, van Putten JW, Maring JG, Zdzienicka MZ, Groen HJ, Kampinga HH (2003). Selective targeting of homologous DNA recombination repair by gemcitabine. International journal of radiation oncology, biology, physics.

[27] Sun Z, Asmann YW, Kalari KR, Bot B, Eckel-Passow JE, Baker TR (2011). Integrated Analysis of Gene Expression, CpG Island Methylation, and Gene Copy Number in Breast Cancer Cells by Deep Sequencing. PloS one.

[28] Geiss GK, Bumgarner RE, Birditt B, Dahl T, Dowidar N, Dunaway DL (2008). Direct multiplexed measurement of gene expression with color-coded probe pairs. Nat Biotechnol.

[29] Malkov VA, Serikawa KA, Balantac N, Watters J, Geiss G, Mashadi-Hossein A (2009). Multiplexed measurements of gene signatures in different analytes using the Nanostring nCounter Assay System. BMC Res Notes.

[30] Reis PP, Waldron L, Goswami RS, Xu W, Xuan Y, Perez-Ordonez B (2011). mRNA transcript quantification in archival samples using multiplexed, color-coded probes. BMC biotechnology.

[31] Veldman-Jones MH, Brant R, Rooney C, Geh C, Emery H, Harbron CG (2015). Evaluating Robustness and Sensitivity of the NanoString Technologies nCounter Platform to Enable Multiplexed Gene Expression Analysis of Clinical Samples. Cancer research.

[32] Kulkarni MM (2011). Digital multiplexed gene expression analysis using the NanoString nCounter system. Curr Protoc Mol Biol.

[33] Martin CL, Reshmi SC, Ried T, Gottberg W, Wilson JW, Reddy JK (2008). Chromosomal imbalances in oral squamous cell carcinoma: examination of 31 cell lines and review of the literature. Oral oncology.

[34] White JS, Weissfeld JL, Ragin CC, Rossie KM, Martin CL, Shuster M (2007). The influence of clinical and demographic risk factors on the establishment of head and neck squamous cell carcinoma cell lines. Oral oncology.

[35] Gottipati P, Vischioni B, Schultz N, Solomons J, Bryant HE, Djureinovic T (2010). Poly(ADP-ribose) polymerase is hyperactivated in homologous recombination-defective cells. Cancer research.

[36] Bryant HE, Schultz N, Thomas HD, Parker KM, Flower D, Lopez E (2005). Specific killing of BRCA2-deficient tumours with inhibitors of poly(ADP-ribose) polymerase. Nature.

[37] Farmer H, McCabe N, Lord CJ, Tutt AN, Johnson DA, Richardson TB (2005). Targeting the DNA repair defect in BRCA mutant cells as a therapeutic strategy. Nature.

[38] Rugo H, Olopade O, DeMichele A, Van't Veer L, Buxton M, Hylton N (2013). Veliparib/carboplatin plus standard neoadjuvant therapy for high-risk breast cancer: First efficacy results from the I-SPY 2 TRIAL. 2013. San Antonio Breast Cancer Symposium.

[39] Mehta M, Curran W, Wang D, Wang F, Kleinberg L, Brade A (2012). Phase I safety and pharmacokinetic (PK) study of veliparib in combination with whole brain radiation therapy (WBRT) in patients (pts) with brain metastases. Journal of clinical oncology : official journal of the American Society of Clinical Oncology.

[40] Bonner JA, Harari PM, Giralt J, Azarnia N, Shin DM, Cohen RB (2006). Radiotherapy plus cetuximab for squamous-cell carcinoma of the head and neck. The New England journal of medicine.

[41] Rosenthal EL, Kulbersh BD, King T, Chaudhuri TR, Zinn KR (2007). Use of fluorescent labeled anti-epidermal growth factor receptor antibody to image head and neck squamous cell carcinoma xenografts. Molecular cancer therapeutics.

[42] Cooper T RM, Trummell HQ, Weaver A, Bonner JA, Yang ES (2013). PARP inhibition in HPV positive head and neck cancers. Cancer research.

[43] Dok R, Kalev P, Van Limbergen EJ, Asbagh LA, Vazquez I, Hauben E (2014). p16INK4a impairs homologous recombination-mediated DNA repair in human papillomavirus-positive head and neck tumors. Cancer research.

[44] Park JW, Nickel KP, Torres AD, Lee D, Lambert PF, Kimple RJ (2014). Human papillomavirus type 16 E7 oncoprotein causes a delay in repair of DNA damage. Radiother Oncol.

[45] Shin KH, Ahn JH, Kang MK, Lim PK, Yip FK, Baluda MA (2006). HPV-16 E6 oncoprotein impairs the fidelity of DNA end-joining via p53-dependent and -independent pathways. International journal of oncology.

[46] Iftner T, Elbel M, Schopp B, Hiller T, Loizou JI, Caldecott KW (2002). Interference of papillomavirus E6 protein with single-strand break repair by interaction with XRCC1. The EMBO journal.

[47] Fragkos M, Jurvansuu J, Beard P (2009). H2AX is required for cell cycle arrest via the p53/p21 pathway. Molecular and cellular biology.

[48] Sewell A, Brown B, Biktasova A, Mills GB, Lu Y, Tyson DR (2014). Reverse-phase protein array profiling of oropharyngeal cancer and significance of PIK3CA mutations in HPV-associated head and neck cancer. Clinical cancer research : an official journal of the American Association for Cancer Research.

[49] Moeller BJ, Yordy JS, Williams MD, Giri U, Raju U, Molkentine DP (2011). DNA repair biomarker profiling of head and neck cancer: Ku80 expression predicts locoregional failure and death following radiotherapy. Clinical cancer research : an official journal of the American Association for Cancer Research.

[50] Gillison ML, Zhang Q, Jordan R, Xiao W, Westra WH, Trotti A (2012). Tobacco smoking and increased risk of death and progression for patients with p16-positive and p16-negative oropharyngeal cancer. Journal of clinical oncology : official journal of the American Society of Clinical Oncology.

[51] Hegan DC, Lu Y, Stachelek GC, Crosby ME, Bindra RS, Glazer PM (2010). Inhibition of poly(ADP-ribose) polymerase down-regulates BRCA1 and RAD51 in a pathway mediated by E2F4 and p130. Proceedings of the National Academy of Sciences of the United States of America.

[52] Boboila C, Jankovic M, Yan CT, Wang JH, Wesemann DR, Zhang T (2010). Alternative end-joining catalyzes robust IgH locus deletions and translocations in the combined absence of ligase 4 and Ku70. Proceedings of the National Academy of Sciences of the United States of America.

[53] Yan CT, Boboila C, Souza EK, Franco S, Hickernell TR, Murphy M (2007). IgH class switching and translocations use a robust non-classical end-joining pathway. Nature.

[54] Tobin LA, Robert C, Nagaria P, Chumsri S, Twaddell W, Ioffe OB (2012). Targeting abnormal DNA repair in therapy-resistant breast cancers. Molecular cancer research : MCR.

[55] Tobin LA, Robert C, Rapoport AP, Gojo I, Baer MR, Tomkinson AE (2013). Targeting abnormal DNA double-strand break repair in tyrosine kinase inhibitor-resistant chronic myeloid leukemias. Oncogene.

[56] Knowles JA, Golden B, Yan L, Carroll WR, Helman EE, Rosenthal EL (2011). Disruption of the AKT pathway inhibits metastasis in an orthotopic model of head and neck squamous cell carcinoma. The Laryngoscope.

[57] Nowsheen S, Cooper T, Stanley JA, Yang ES (2012). Synthetic lethal interactions between EGFR and PARP inhibition in human triple negative breast cancer cells. PloS one.

