# Supplementary material for: DNA double strand break repair defect and sensitivity to poly ADP-ribose polymerase (PARP) inhibition in human papillomavirus 16-positive head and neck squamous cell carcinoma
Source: Oncotarget. 2015 Aug 22;6(29):26995–7007. doi: 10.18632/oncotarget.4863 (PMC4694969; doi:10.18632/oncotarget.4863)
Supplement: Supplementary file 1 [file oncotarget-06-26995-s001.pdf]

SUPPLEMENTARY FIGURES AND TABLE

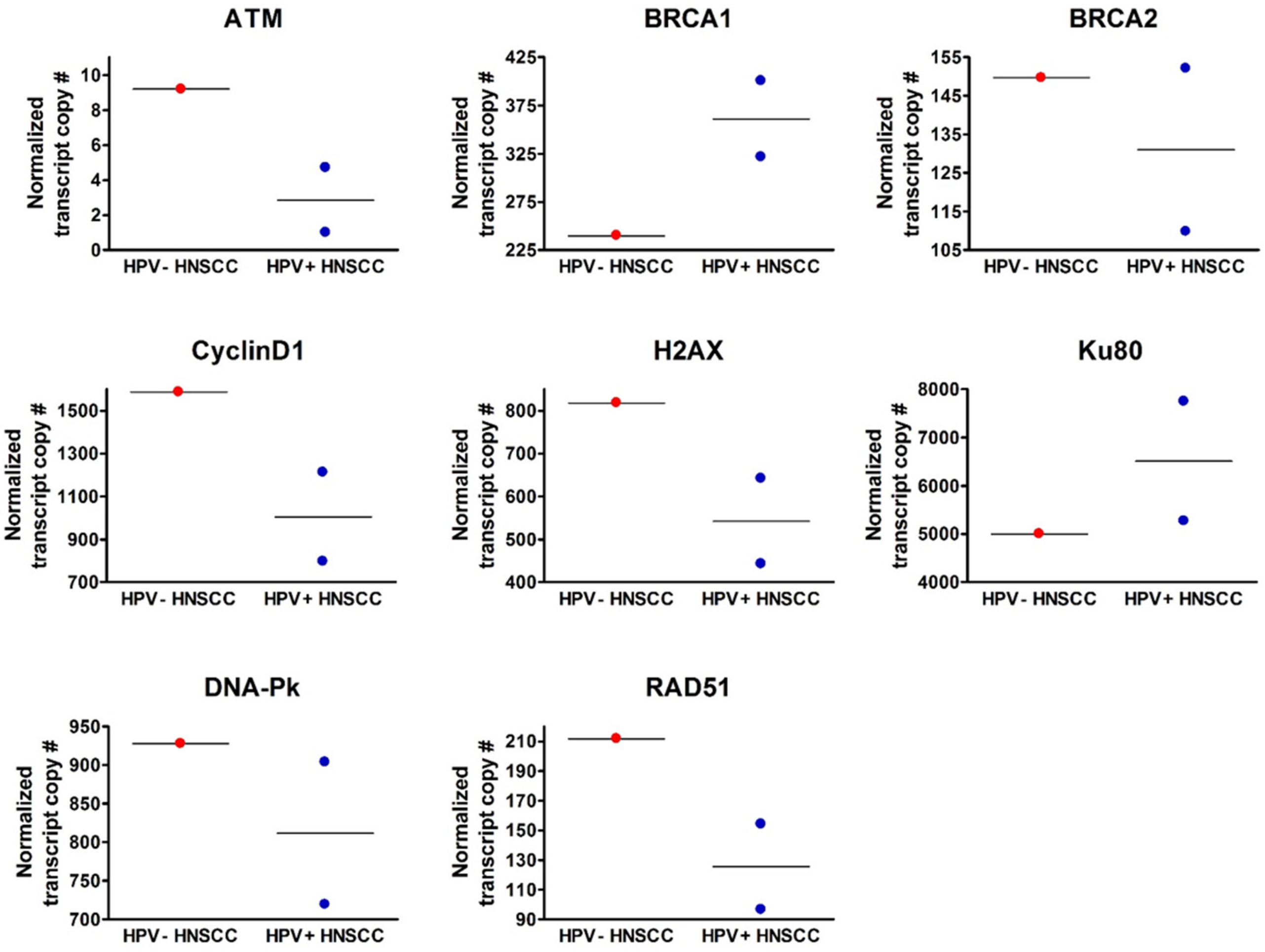

Supplementary Figure S1: mRNA expression of DSB repair genes in HNSCC cell lines. RNA was extracted from UM-SCC1, UM-SCC47, and UPCI:SCC154 cells and processed using the NanoString nCounter platform. Gene expression data for each cell line were normalized using a built-in panel of housekeeping genes. Shown are normalized transcript copy numbers per 100 ng of RNA.

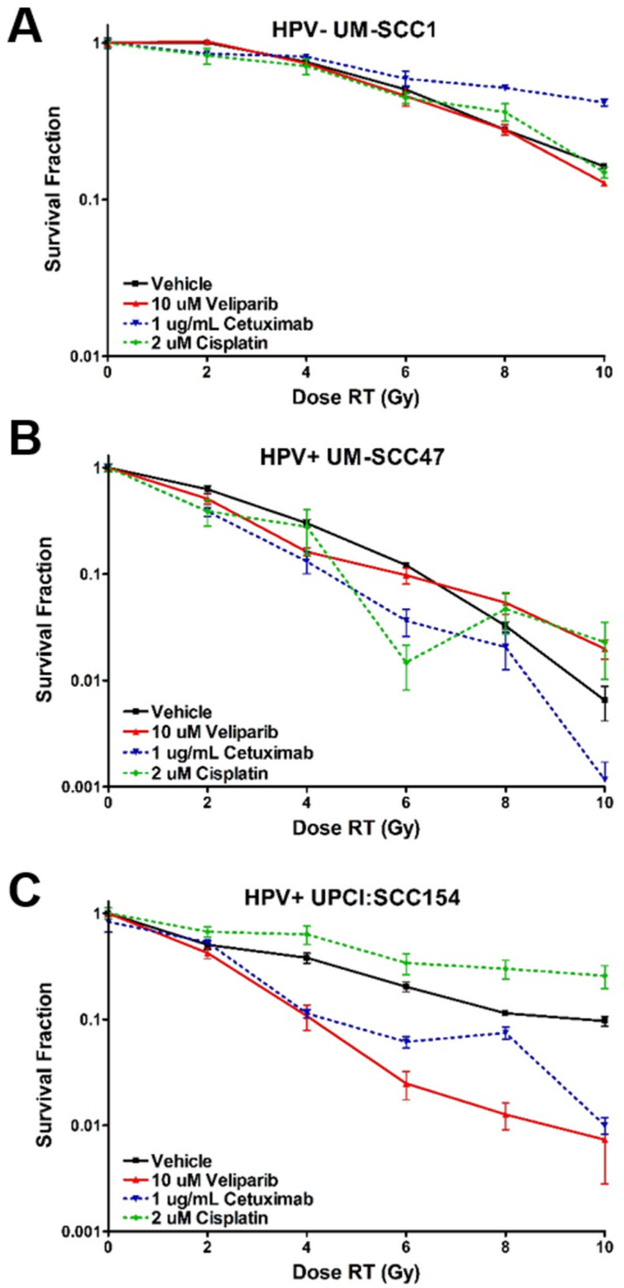

Supplementary Figure S2: Veliparib, cetuximab, and cisplatin as radiosensitizers in HNSCC cell lines. A. UM-SCC1, **B.** UM-SCC47, and **C.** UPI:SCC154 cells were plated at two different densities and treated with the indicated doses of the PARP inhibitor veliparib, the anti-EGFRr monoclonal antibody cetuximab, or cisplatin in combination with increasing doses of IR. After treatment, cells were left undisturbed for approximately 2 weeks, then fixed and stained for colony counting. Shown is the mean +/− SEM from at least 2 independent experiments performed in triplicate.

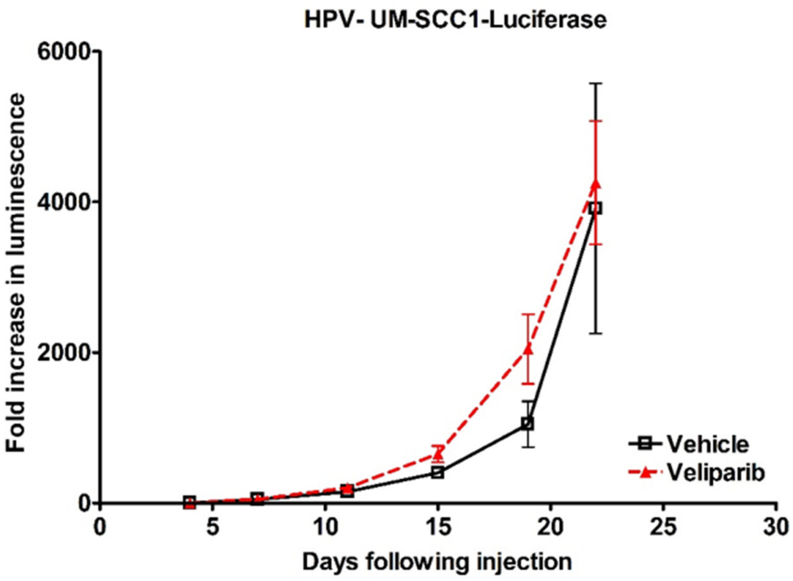

Supplementary Figure S3: HPV- HNSCCs are not sensitive to PARP inhibition *in vivo*. 100, 000 UM-SCC1-luciferase cells were injected orthopically into NOD-SCID mice. Tumor volume was calculated bi-weekly using a luciferase bioluminescence assay. At post-injection day 7, mice were randomly divided into vehicle (normal saline) or veliparib (200 mg/kg twice daily) groups with 10 mice per group. Mice were treated by oral gavage twice per day. Shown is the mean fold change in tumor volume +/− SEM. ***p* < 0.01.

Supplementary Table S1: HNSCC cell line characteristics

Cell Line	Site of Origin	HPV16 Status	Viral Load	Smoking Status	p53 Status
UM-SCC1	Oral cavity	Negative	0	Unknown	Wild type, no expression
UM-SCC47	Oral cavity	Positive, integrated	15	Positive	Exon 4 polymorphism, weak expression
UPCI:SCC154	Oral cavity	Positive, integrated	1	Positive	Wild type
